# New Perspectives on the Immunopathogenesis and Treatment of Uveitis Associated With Vogt-Koyanagi-Harada Disease

**DOI:** 10.3389/fmed.2021.705796

**Published:** 2021-11-12

**Authors:** Ahmed M. Abu El-Asrar, Jo Van Damme, Sofie Struyf, Ghislain Opdenakker

**Affiliations:** ^1^Department of Ophthalmology, College of Medicine, King Saud University, Riyadh, Saudi Arabia; ^2^Dr. Nasser Al-Rashid Research Chair in Ophthalmology, College of Medicine, King Saud University, Riyadh, Saudi Arabia; ^3^Laboratory of Molecular Immunology, Department of Microbiology, Immunology and Transplantation, Rega Institute for Medical Research, University of Leuven, Leuven, Belgium; ^4^Laboratory of Immunobiology, Department of Microbiology, Immunology and Transplantation, Rega Institute for Medical Research, University of Leuven, Leuven, Belgium

**Keywords:** autoimmune disease, B cells, immunomodulatory therapy, therapeutic window of opportunity, remnant epitope, molecular mimicry

## Abstract

Uveitis associated with Vogt-Koyanagi-Harada (VKH) disease is a bilateral, chronic, granulomatous autoimmune disease associated with vitiligo, poliosis, alopecia, and meningeal and auditory manifestations. The disease affects pigmented races with a predisposing genetic background. Evidence has been provided that the clinical manifestations are caused by a T-lymphocyte-mediated autoimmune response directed against antigens associated with melanocytes in the target organs. Alongside of T lymphocytes, autoreactive B cells play a central role in the development and propagation of several autoimmune diseases. The potential role of B lymphocytes in the pathogenesis of granulomatous uveitis associated with VKH disease is exemplified within several studies. The early initial-onset acute uveitic phase typically exhibits granulomatous choroiditis with secondary exudative retinal detachment and optic disc hyperemia and swelling, subsequently involving the anterior segment if not adequately treated. The disease eventually progresses to chronic recurrent granulomatous anterior uveitis with progressive posterior segment depigmentation resulting in “sunset glow fundus” appearance and chorioretinal atrophy if not properly controlled. Chronically evolving disease is more refractory to treatment and, consequently, vision-threatening complications have been recognized to occur in the chronic recurrent phase of the disease. Conventional treatment with early high-dose systemic corticosteroids is not sufficient to prevent chronic evolution. Addition of immunomodulatory therapy with mycophenolate mofetil as first-line therapy combined with systemic corticosteroids in patients with acute initial-onset disease prevents progression to chronic evolution, late complications, vitiligo, and poliosis. Furthermore, patients under such combined therapy were able to discontinue treatment without relapse of inflammation. These findings suggest that there is a therapeutic window of opportunity for highly successful treatment during the early initial-onset acute uveitic phases, likely because the underlying disease process is not fully matured. It is hypothesized that early and aggressive immunosuppressive therapy will prevent remnant epitope generation in the initiation of the autoimmune process, the so-called primary response. B cell depleting therapy with the anti-CD20 monoclonal antibody rituximab is effective in patients with refractory chronic recurrent granulomatous uveitis. The good response after rituximab therapy reinforces the idea of an important role of B cells in the pathogenesis or progression of chronic recurrent uveitis associated with VKH disease.

## Introduction

Vogt-Koyanagi-Harada (VKH) disease is a multisystem T-lymphocyte-mediated autoimmune disease directed against antigens associated with melanocytes present in the target organs including the uvea, inner ear, meninges, and integumentary system. Several studies demonstrated that tyrosinase family proteins are important antigens specific to VKH disease (1–3).

**Figure 1:**
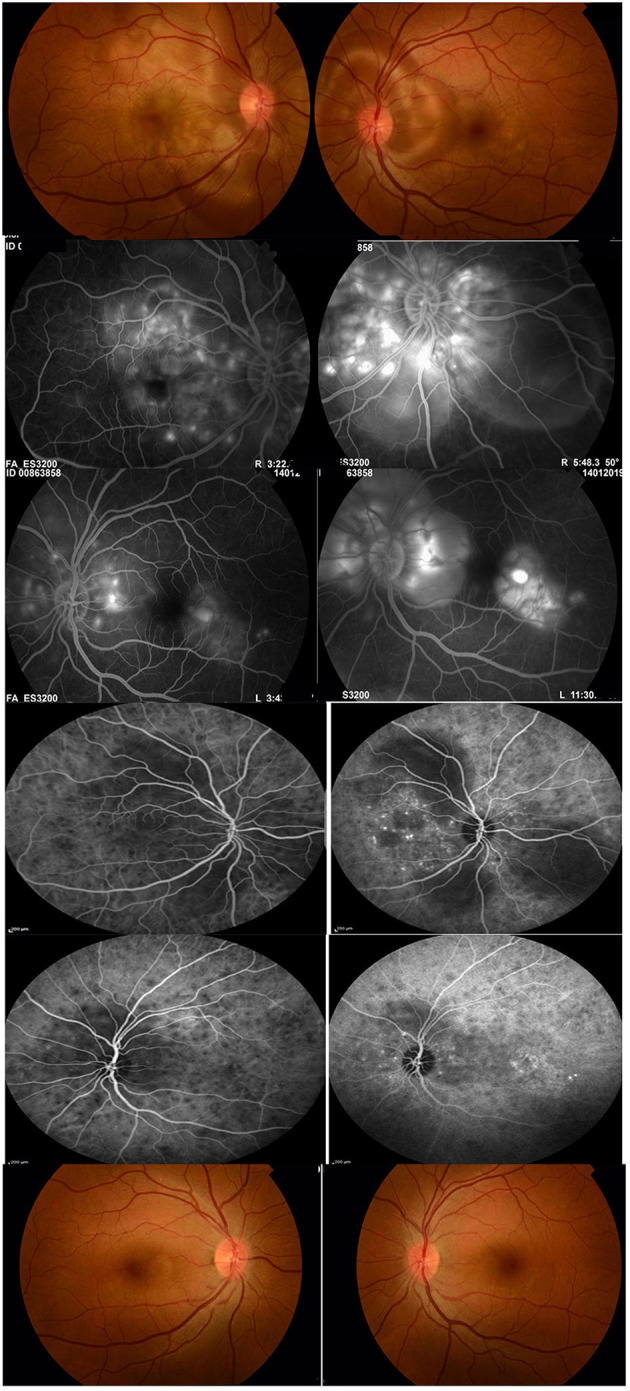
A 36-year-old female with initial-onset acute uveitis associated with Vogt-Koyanagi-Harada disease at presentation. Visual acuity was 20/200 in the right eye and 20/60 in the left eye (first row). Fluoresceine angiography shows multiple pinpoint hyperfluorescent spots at the level of the retinal pigment epithelium, late pooling of dye in the areas of exudative retinal detachment (second and third rows). Indocyanine green angiography shows multiple hypofluorescent spots corresponding to choroidal granulomas, hypofluorescent patches corresponding to areas of exudative retinal detachment and punctate choroidal hyperfluorescence (fourth and fifth rows). The patient received systemic corticosteroids combined with mycophenolate mofetil. Twenty-six months after treatment, best visual acuity was 20/20 in both eyes. The patient was off treatment for 10 months without relapse of inflammation. Note the absence of “sunset glow fundus” and of chorioretinal atrophy (sixth row).

**Figure 2:**
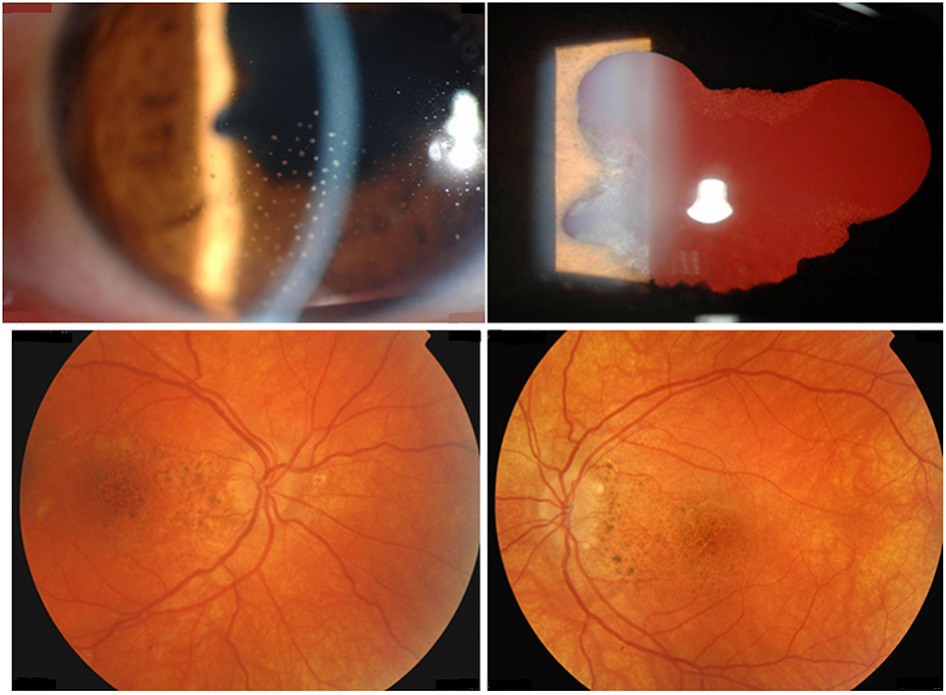
A 39-year-old female with chronic recurrent uveitis associated with Vogt-Koyanagi-Harada disease at presentation. Slit-lamp biomicroscopy shows mutton-fat granulomatous keratic precipitates (first row). Fundus photography shows posterior segment depigmentation resulting in “sunset glow fundus” (second row).

Patients with VKH disease show different clinical manifestations depending on the duration of the disease before presentation. Patients with initial-onset acute uveitis present with granulomatous choroiditis with secondary exudative retinal detachment resulting from impairment of the retinal pigment epithelium caused by choroidal inflammation with typical optic nerve head swelling and hyperemia (Figure 1). During the acute uveitic phase, compression of the vessels by edema and granulomatous inflammation interferes with choroidal blood flow and induces choroidal circulation impairment. Laser speckle flowgraphy studies demonstrated inflammation-related impairment of choroidal and optic nerve head blood flow velocity in patients with acute uveitis associated with VKH disease. Systemic immunosuppressive therapy improved inflammation-related impairment in blood flow velocity (4). These circulatory disturbances may cause axonal flow stasis and secondary axonal swelling of the optic nerve head (5). The inflammation subsequently involves the anterior segment if not adequately treated. The disease will proceed to chronic recurrent granulomatous anterior uveitis if not properly controlled (6–8). Progressive subclinical choroidal inflammation due to inadequate immunosuppression in the initial-onset acute uveitic phase will lead to progressive posterior segment depigmentation resulting in “sunset glow fundus” appearance and chorioretinal atrophy (Figure 2) (9–11). During the acute phase, systemic manifestations include neurological and auditory signs. Neurological manifestations include meningismus (headache and stiffness of the neck and back) with cerebrospinal fluid (CSF) lymphocytic pleocytosis. It is presumed that pleocytosis is caused by cell-mediated immunoreaction against melanocytes in the meninges (12). The audiovestibular symptoms include sensorineural hearing loss, tinnitus, and vertigo. In the chronic recurrent phase, integumentary manifestations (poliosis, vitiligo, and alopecia) may develop. The disease affects pigmented races and individuals of certain genetic predisposition (13). HLA-DR4 and HLA-DRW_53_, with the most significant risk allele being HLA-DRB1^*^0405, were found to be genetically associated with VKH disease (7).

Patients with chronic recurrent disease manifest more severe granulomatous inflammation of the anterior segment at presentation. In addition, final visual acuity and mean retinal sensitivity are worse in chronic recurrent disease compared with initial-onset acute disease (8, 14). Studies using laser flare-cell meter demonstrated that both aqueous flare values and cell counts were significantly higher in chronic recurrent disease than those in initial-onset acute disease. Moreover, chronic recurrent granulomatous inflammation is more refractory to treatment, needing prolonged and repeated therapeutic interventions (15). Therefore, vision-threatening complications including glaucoma, cataract, subretinal fibrosis, chorioretinal atrophy, choroidal neovascular membranes, and “sunset glow fundus,” are more common in chronic recurrent disease (6, 8, 9). Indocyanine green angiographic studies of patients during episodes of apparent isolated granulomatous anterior segment recurrence demonstrated choroidal involvement suggesting ongoing subclinical choroidal inflammation despite the absence of clinical signs of posterior segment inflammation (16). Histopathologic studies of eyes with “sunset glow fundus” revealed the presence of scattered inflammatory infiltrates in the thickened choroid with disappearance of choroidal melanocytes (17). These findings suggest that ongoing subclinical choroidal inflammation due to inadequate immunosuppression is involved in the pathogenesis of progressive posterior segment depigmentation resulting in “sunset glow fundus” appearance and chorioretinal atrophy (16–19). Therefore, the main goals in the treatment of the acute uveitic phase are to suppress the uveal inflammation in the acute posterior uveitis stage with adequate immunosuppressive therapy and prevention of the progression to chronic recurrent evolution.

This article aims to provide new insights on the immunopathogenesis and to open perspectives for treatment of uveitis associated with VKH disease.

## Defining The Therapeutic Window of Opportunity in Initial-Onset Acute Uveitis Associated With VKH Disease

A therapeutic window of opportunity is a well-established concept of critical importance in rheumatoid arthritis clinical research and considerable attention has been paid to when it might close. This window represents a very early phase of the disease in which therapeutic disease modification is more successful, presumably because of a not fully matured underlying disease process (20). A growing body of evidence has emphasized the consistent clinical and radiographic benefits of prompt initiation of disease-modifying antirheumatic drugs in patients with rheumatoid arthritis during early stages of the disease. Therapeutic intervention in this period alters the natural history of the disease and hampers disease progression in such a way that chronicity is reduced. These findings have led to changes in rheumatoid arthritis treatment paradigms, with increasing emphasis on early diagnosis and treatment (21, 22).

In a prospective study, it was demonstrated that none of the patients who presented early with initial-onset acute VKH disease without anterior segment inflammation progressed to chronic recurrent disease or developed any complication of cataract or glaucoma or subretinal neovascular membranes or “sunset glow fundus” or chorioretinal atrophy during the follow-up period. In contrast, 16.4 and 13.6% of the patients with initial-onset acute VKH disease with anterior segment inflammation due to delayed patient presentation developed glaucoma and cataract, respectively, during the follow-up period (9). Such findings are suggestive for a need of prompt diagnosis and therapy without delay in the very early phases of acute VKH disease. It seems that prompt and adequate treatment before the occurrence of anterior segment inflammation is essential to prevent the development of complications. In a similar way to rheumatoid arthritis, catching this therapeutic window of opportunity in the very early phases of acute uveitis associated with VKH disease prevents progression to chronic recurrent evolution and development of complications and “sunset glow fundus.” This window represents a very early disease phase in which therapeutic disease modification is more successful, likely because the underlying disease process is not fully matured (20–22).

## Systemic Corticosteroid Monotherapy is not Effective in Preventing Chronic Recurrent Evolution and Development of Complications and “Sunset Glow Fundus”

Early diagnosis, timely initiation of immunosuppressive therapy and appropriate and adequate treatment are key to the optimal management of uveitis associated with VKH disease. A delay in diagnosis and in initiating adequate treatment are associated with a high risk of developing disease chronicity, complications and visual impairment (23).

Increasing evidence suggests that, despite proper treatment with corticosteroid monotherapy, many patients progress to chronic recurrent granulomatous inflammation and develop “sunset glow fundus” and chorioretinal atrophy even after the clinical disease appears to be under control (23). Keino et al. (24) retrospectively studied 80 patients with VKH disease treated with high-dose systemic corticosteroid therapy from initial-onset. Chronic ocular inflammation developed in 17.5% of patients, and “sunset glow fundus” developed in 67.5% of these patients. In another study, Keino et al. (25) reviewed the charts of 102 patients with VKH disease who were treated with high-dose systemic corticosteroid therapy at initial onset. “Sunset glow fundus” was observed in 67.6% of these patients. The mean duration until the appearance of “sunset glow fundus” was 4.2 ± 2.7 months. Chee et al. (26) observed that one-third of patients receiving high-dose systemic corticosteroid therapy within 2 weeks of presenting with symptoms progressed to chronic recurrent disease. Tugal-Tutkun et al. (27) observed that 95% of patients who presented in the acute uveitic phase progressed to chronic recurrent evolution. Lai et al. (18) demonstrated the development of “sunset glow fundus” in 51.4% of patients who received systemic corticosteroids during the first attack of VKH disease. Sakata et al. (28) demonstrated that in spite of early high-dose systemic corticosteroid therapy within 30 days from disease onset and a slow taper, 79% of patients with VKH disease progressed to chronic recurrent evolution, and 38% developed subretinal fibrosis. Recently, Nakayama et al. (29) demonstrated that despite high-dose corticosteroid therapy in patients with new-onset acute VKH disease, recurrent inflammation was observed in 22.5% of patients. In this cohort, “sunset glow fundus” developed in 49.5% of eyes and ocular complications were observed in 21.2% of eyes.

## Immunomodulatory Therapy as First-Line Treatment Combined With Systemic Corticosteroids in Initial-Onset Acute Uveitis Associated with VKH Disease Prevents Chronic Recurrent Evolution and Cures the Disease

Because of the poor prognosis associated with chronic recurrent evolution and the well-documented complications of long-term high-dose corticosteroid treatment, several retrospective studies suggested to initiate non-steroidal immunomodulatory therapy in addition to systemic corticosteroids early in the course of the disease to achieve better control of the uveitis and to facilitate earlier tapering of corticosteroids (7, 8, 30–32). In prospective studies, addition of immunomodulatory therapy with mycophenolate mofetil as first-line therapy combined with systemic corticosteroids in patients with initial-onset acute uveitis associated with VKH disease prevented the progression of the disease to chronic recurrent evolution and development of complications and “sunset glow fundus.” Furthermore, mycophenolate mofetil was effective in preventing the development of vitiligo, poliosis, alopecia and sensory hearing loss (10, 11). These findings suggest that mycophenolate mofetil was effective in controlling progressive subclinical choroidal inflammation. Therefore, uveal depigmentation and chorioretinal atrophy should not be regarded as signs of convalescence. Rather, we suggest that they be considered signs of ongoing subclinical choroidal inflammation due to inadequate immunosuppressive therapy. One of the most important goals in using non-steroidal immunomodulatory treatment in non-infectious uveitis is to minimize exposure to corticosteroids and to reduce corticosteroid levels. In our study, a corticosteroid-sparing effect (prednisone dose ≤ 10 mg/day) was achieved in all patients after a mean time of 3.8 ± 1.3 months. Furthermore, patients were able to discontinue treatment without relapse of inflammation (Figure 1) (11).

These findings suggest that, similar to rheumatoid arthritis, there is a therapeutic window of opportunity for highly successful treatment of uveitis associated with VKH disease during the very early acute uveitic phase, likely because the underlying disease process is not fully matured (20–23). Treatment with mycophenolate mofetil as first-line therapy combined with systemic corticosteroids during the therapeutic window is able to modify the phenotype of VKH disease and leads to substantial improvement of disease outcome avoiding chronic evolution, “sunset glow fundus,” late complications, vitiligo, poliosis, alopecia, and sensory hearing loss and it achieved cure of disease after discontinuation of treatment.

Recently, Lin Oo et al. (33) showed that despite introducing immunodulatory therapy with azathioprine in most of the patients within the first 3 months of onset of VKH disease combined with systemic corticosteroids, chronic evolution occurred in 51.7% of the eyes and “sunset glow fundus” developed in 58.6% of the eyes. They suggested that timing of initiation of immunomodulatory therapy is a key factor for success of treatment as immunomodulatory therapy does not take effect immediately, often taking months to have maximal effect. They too, therefore, recommended introducing immunomodulatory therapy as first-line therapy combined with systemic corticosteroids rather than when the dose of corticosteroids has been decreased.

## VKH Disease is a Prototypic Example of Autoimmune Disease

To distinguish autoimmune from (auto)inflammatory diseases clear definitions and criteria are applicable and these relate to (i) the characterization of specific autoantigens, (ii) a chronic disease course, and (iii) the involvement of adaptive immune cells and molecules, namely T and B lymphocytes with their antigen-specific T cell receptors and antibodies (20). The classical two-signal paradigm for adaptive immune activation against infections entails the antigenic stimulus as “signal 1” and additional molecules, mainly cell adhesion molecules and cytokines as “signal 2” and this provides an immune activation mode when needed and represents a safeguard against autoimmunity (34). From a mechanistic point of view, in autoimmune diseases this “signal 1” is an autoantigen and an infection or an inflammation constitute the environmental condition for induction of the necessary cytokines and adhesion molecules to provide “signal 2.” These aspects may be recapitulated by the development of preclinical animal models, that not only help to understand the immunopathogenesis but also to study therapeutic options. Refined techniques to analyze human genetic details and immune repertoires assist in better diagnosis, susceptibility profiling, prognosis and therapy of VKH disease patients. VKH disease is a true autoimmune disease with primarily tyrosinase and tyrosinase-related protein-1 and −2 (TRP-1, TRP-2) being known autoantigens in humans (1) and rats (2). At some point in ontogeny, a trigger leads to activation of specific subsets of T lymphocytes. Most probably, pro-inflammatory Th1 and Th17 cells are triggered by antigens presented within the context of major histocompatibility complex (MHC) class II (HLA-DRB1^*^04:05). The latter HLA class II antigen is known as an important genetic susceptibility factor in VKH disease (7, 35). Melanocyte-specific peptides have been identified as autoantigenic “signal 1” with the use of functional epitope scanning analysis (36). Future crystallography and cryo-electron microscopy studies may elucidate how autoimmune host peptides are presented in HLA-DRB1^*^04:05 and how these structural details ensure the interaction with and activation of T cells with a specific T cell receptor. Although the identification of VKH disease-related autoantigens by T cell epitope scanning in individual patients characterizes these persons as having an autoimmune disease, clinical cases exist in which the presence of the above-mentioned autoantigens was difficult to proof (37). Therefore, it remains possible that additional autoantigens for activation of T cells are at play to induce cytotoxicity by CD8-positive T cells or CD4-positive helper cell activities for autoantibody induction. Alternatively, stimulation of specific B lymphocytes from the primary repertoire as immunogenic “signal 1” in the presence of an infection, e.g., by a herpes virus, as signal 2 for helper activity induction as bystander effect, may lead to autoantibody formation (*vide infra*). Both antibodies and autoreactive T cells against autoantigens cause autoimmune-related cytotoxicity and tissue damage.

Caution and a critical attitude about causal inference remain necessary about infections as triggers for VKH disease (38). Virus infections, including Hepatitis B (39), Hepatitis C virus under interferon therapy (40), cytomegalovirus (CMV) and other herpesviruses (38, 41) have all been associated with VKH disease. Efforts to corroborate the latter findings failed so far and the ensemble of such studies cautions that an association does not necessarily represent a cause (42). In this respect, the type of body fluid analyzed (vitreous vs. cerebrospinal fluid) may be critical to detect the presence of viruses, as it is known that even in single patients different strains of viruses may be compartmentalized at specific locations (37, 43).

**Figure 3:**
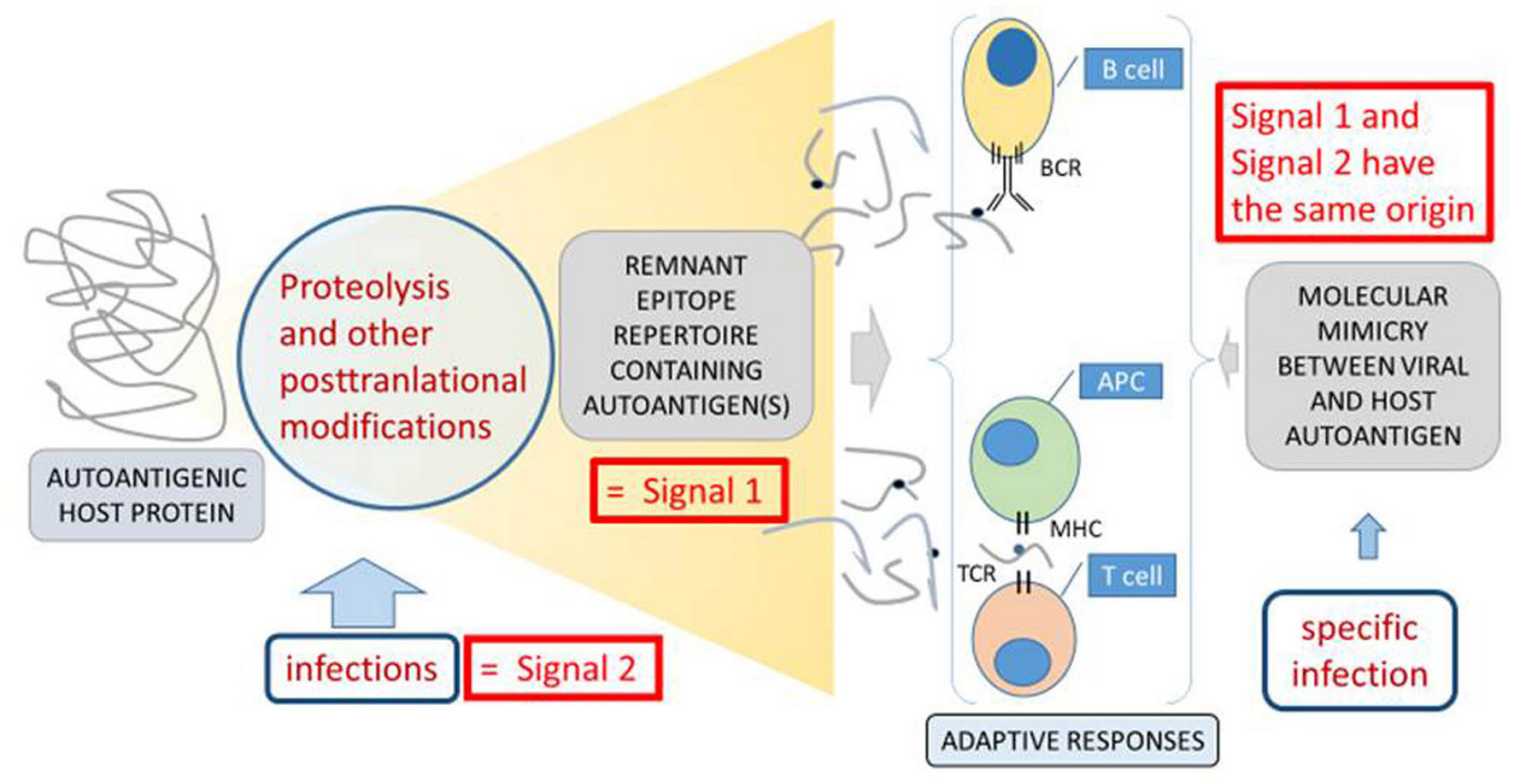
Molecular mimicry, remnant epitopes and infections in VKH disease, as an example of autoimmunity. Two basic mechanisms of autoimmunity, molecular mimicry indicated at the right side (44) and remnant epitopes indicated at the left side (20) are compared. For the development of adaptive immune responses (indicated centrally at the bottom and between double braces), two signals are necessary (34). The so-called “signal 1” is the antigenic stimulus, which in the cases of autoimmune processes is an autoantigen, eliciting B cell or T cell responses through their cognate receptors (BCR and TCR). “Signal 2” is in most cases an environmental signal from an infection, which is translated at the molecular level into cell adhesion molecules or cytokines as reinforcers of the actions of the immunological synapse between an antigen-presenting cell (APC) and a T cell or in the activation of B cells. In the situation of molecular mimicry (44), the infection itself provides both the antigenic stimulus and the activation of adaptive immune cells. In this case “Signal 1” and “Signal 2” have the same origin and structural resemblance between a host autoantigen and a microbial epitope leads to cross-reactive “signal 1.” In the cases of remnant epitopes, various forms of infections may provide direct proteolysis and posttranslational modifications or induce host proteases and other enzymes. Their concerted actions lead to the generation of collections of autoantigenic peptides with an enhanced propensity to provide “signal 1,” when compared to healthy conditions. When this happens in the context of an infection or inflammation, the required “signal 2” is also present. Figure adapted from reference (20).

Virus infections may provide direct or indirect triggers to initiate or perpetuate VKH disease at least in two different ways. First and as hypothesized by the molecular mimicry paradigm (44), linear or conformational viral protein antigens from e.g., CMV may cross-react with tyrosinase peptides in T cell activation tests (45). Secondly and as hypothesized by the remnant epitope paradigm (20) extracellular inflammatory proteolysis, associated with various types of viral or bacterial infections, may locally lead to degradation of melanocyte proteins into remnant epitopes. On a statistical basis (cleavage sites, abundancy class) the generated autoantigenic peptides enhance the chance of (re)activation of autoreactive T or B lymphocytes. In both paradigms, molecular mimicry and remnant epitopes, the two necessary signals for T cell activation (remnant epitope or viral antigen as signal 1 and the infectious/inflammatory context as signal 2) are both simultaneously present (Figure 3). So far, it is easier to understand how the two mechanisms of mimicry and remnant epitopes play a role in the reactivation than in the initiation of VKH disease. It is also conceivable that both paradigms are at play together in the autoimmune initiation process. In view of observed frequencies of occurrence of autoimmune diseases, it is plausible that remnant epitopes (associated with various types of infectious agents) are more common than molecular mimicry (associated with a single infection). Finally, it may be questioned how intracellular molecules, such as tyrosinase or TRP-1 and TRP-2, may be cleaved by extracellular proteases to yield immunogenic remnant epitopes in the autoimmune process of VKH disease. Various processes leading to different types of cytolysis may be invoked when infections and collateral cell and tissue damage develop. For instance, punctuate lesions observed in VKH patients *in vivo* and named mutton-fat granulomatous keratic precipitates (as visualized in Figure 2) are reminiscent of a virus plaque assay *in vitro* and may indicate spots where cytolysis occurs, either by the direct cytopathogenic effect of a virus or by various types of indirectly induced forms of immune-mediated cytotoxicity. For instance, the classical activation pathway of the complement system by immune complexes or the action of cytotoxic T lymphocytes or natural killer cells may lead to host cell cytolysis. As an additional possibility, also intracellular proteases, such as caspases, may generate remnant epitopes from intracellular substrates, such as tyrosinase and TRP-1 and −2 and these may become accessible for antigen presentation to T cells and for B lymphocytes when the host cell disintegrates, e.g., by apoptosis.

## B Lymphocytes in the Pathogenesis of Granulomatous Uveitis Associated With VKH Disease

Analysis of the aqueous humor samples from patients with uveitis may help in generating novel biomarkers for different phenotypes of uveitis. This type of information may be helpful for better diagnosis and to improve treatment by better stratification of patients. Currently, a commonly used approach to enhance specificity and selectivity for diagnostic purposes is to combine various markers. Aside this, the discovery of pathogenic biomarkers is preferred, notwithstanding that it may be more laborious. Only after the causal role of specific molecules for particular disease entities is established, these may serve as potential targets for selective therapy.

Toward the aim of finding pathogenic biomarkers for various clinical entities of uveitis, aqueous humor samples from patients with active uveitis associated with Behçet's disease, sarcoidosis, HLA-B27-related intraocular inflammation and VKH disease were compared for the presence of chemokines, cytokines and soluble cytokine receptors (46–51). Results of pairwise comparisons are shown in Table 1. Although for VKH disease the number of possible biomarkers was frustratingly low: two stood out as possibly relevant and both were related to B cell biology: CXCL13 and the tumor necrosis factor (TNF)-like weak inducer of apoptosis (TWEAK) (46–51).

**Table 1:** Summary of pairwise comparisons between disease groups.

**I. CXC chemokines**
A. Neutrophil chemoattractants
• Growth-related oncogene (GRO)-α/CXCL1
BD > VKH, sarcoidosis
HLA-B27 > VKH, sarcoidosis
• GRO-β/CXCL2
BD > VKH
HLA-B27 > VKH, sarcoidosis
• Epithelial-derived neutrophil attractant-78 (ENA-78)/CXCL5
BD > VKH
HLA-B27 > VKH, sarcoidosis
• Granulocyte chemotactic protein-2 (GCP-2)/CXCL6
BD > VKH
HLA-B27 > VKH, sarcoidosis
• Interleukin-8 (IL-8)/CXCL8
BD > VKH
HLA-B27 > VKH, sarcoidosis
B. T helper-1 (Th1) lymphocyte chemoattractants
Interferon-γ-inducible protein of 10 kDa (IP-10)/CXCL10
BD >VKH
C. Lymphoid chemokines
B cell attracting chemokine-1 (BCA-1)/CXCL13
VKH, sarcoidosis > BD
VKH > HLA-B27
**II. Soluble form of transmembrane chemokines (Preferentially act on Th1 lymphocytes)**
• Scavenger receptor for phosphatidyl serine and oxidized low-density lipoprotein
(SR-PSOX)/CXCL16
BD > VKH, sarcoidosis
HLA-B27 > VKH, sarcoidosis
• Fractalkine/CX3CL1
BD > VKH
HLA-B27 > VKH, sarcoidosis
Sarcoidosis > VKH
**III. CC chemokines**
• Monocyte chemotactic protein (MCP)-1/CCL2
BD > VKH
HLA-B27 > VKH, sarcoidosis
• MCP-3/CCL7
BD > VKH
HLA-B27 > VKH, sarcoidosis
• MCP-2/CCL8
BD, HLA-B27 > sarcoidosis
HLA-B27 > VKH
• MCP-4/CCL13
HLA-B27 > VKH, sarcoidosis
• Thymus- and activation-related chemokine (TARC)/CCL17
HLA-B27 > VKH
• Macrophage inflammatory protein (MIP)-3α/CCL20
BD > VKH
HLA-B27 > BD, VKH, sarcoidosis
• Thymus-expressed chemokine (TECK)/CCL25
HLA-B27 > VKH, sarcoidosis
• Eotaxin-3/CCL26
BD > VKH
HLA-B27 > VKH, sarcoidosis
**IV. Cytokines and soluble cytokine receptors**
• Interferon-γ
BD > VKH
HLA-B27 > VKH, sarcoidosis
• Tumor necrosis factor-α (TNF-α)
HLA-B27 > VKH
• Interleukin (IL)-1β
BD > VKH
HLA-B27 > BD, VKH, sarcoidosis
• IL-6
BD > VKH, sarcoidosis
HLA-B27 > VKH, sarcoidosis
• IL-11
HLA-B27 > VKH, sarcoidosis
• IL-12p40
BD > VKH
HLA-B27 > VKH
• IL-19
HLA-B27 > BD, VKH, sarcoidosis
• IL-35
HLA-B27 > VKH
• Granulocyte-macrophage colony-stimulating factor (GM-CSF)
HLA-B27 > VKH, sarcoidosis
• The TNF-like weak inducer of apoptosis (TWEAK)
VKH > HLA-B27
Sarcoidosis > BD, HLA-B27
• Soluble (s) CD30
VKH > BD, HLA-B27

*BD, Behçet's disease; HLA-B27, HLA-B27-associated uveitis; VKH, Vogt-Koyanagi-Harada disease*.

CXCL13 was originally identified as B cell attracting chemokine 1 (52), expressed in lymph follicles and in the liver, spleen, and gut. It assists in the homing of B lymphocytes to these sites via its receptor CXCR5, originally named Burkitt's lymphoma receptor-1 (53). Related to autoimmunity, several complementary studies demonstrated that CXCL13 is an important mediator of human autoimmune disorders, such as rheumatoid arthritis (54, 55), systemic lupus erythematosus (56, 57), multiple sclerosis (58, 59) and myasthenia gravis (60). CXCL13 levels were upregulated in synovial tissue (54) and plasma (55) from patients with rheumatoid arthritis, in serum (56) and plasma (57) from patients with systemic lupus erythematosus, in CSF from patients with multiple sclerosis (58, 59) and in serum from patients with myasthenia gravis (60). These studies identified CXCL13 to be a useful inflammatory biomarker (54–60). CXCL13 neutralizing monoclonal antibody significantly reduced disease severity in animal models of rheumatoid arthritis and experimental autoimmune encephalomyelitis (61).

The levels of the B cell chemoattractant CXCL13 in aqueous humor samples from patients with uveitis associated with VKH disease largely exceeded those in patients with Behçet's disease or HLA-B27-associated uveitis (46, 47). In these studies, the concentrations of CXCL13 in the aqueous humor samples were quantified in two sets of aqueous humor samples with the use of two multiplex assays (46, 47). The levels of CXCL13 were elevated 1423.6-fold in VKH disease, 298.3-fold in Behçet's disease, 107.1-fold in HLA-B27-associated uveitis and 458.2-fold in sarcoidosis compared to controls (47). CXCL13 levels in VKH disease were significantly higher than the levels in Behçet's disease and HLA-B27-associated uveitis. However, CXCL13 levels did not differ significantly between VKH disease and sarcoidosis (47, 48).

Additionally, activities of B cells are orchestrated by members of the TNF superfamily, such as TWEAK, a pro-inflammatory inducing ligand (APRIL) and the B-cell-activating factor of the TNF family (BAFF). Consequently, these cytokines play a critical role in B-cell-driven autoimmune diseases (62). The levels of TWEAK, APRIL and BAFF are elevated in the aqueous humor samples of patients with uveitis associated with VKH disease (48). The increased levels of these cytokines may promote B cell survival, differentiation, proliferation and maturation, immunoglobulin class switching, and antibody production (48). Additionally, the predominance of CD20^+^ B lymphocytes in the choroidal inflammatory cell infiltrate is described in patients with sympathetic ophthalmia, which is pathologically identical to VKH disease (63–65), and VKH disease (17, 66). These findings suggest that B lymphocytes may be pathogenically important in granulomatous uveitis associated with VKH disease and that pathogenic B cell depletion might be effective in patients with refractory granulomatous uveitis associated with VKH disease.

The original finding of autoantibodies against host antigens and the formation of pathogenetic immune complexes (20) has been reinforced with interventional studies, providing increasing evidence that B lymphocytes play an important role in the pathogenesis of autoimmune diseases, such as myasthenia gravis, autoimmune thyroiditis, multiple sclerosis, rheumatoid arthritis, and systemic lupus erythematosus (66–69). In addition to producing autoantibodies, B cells may capture antigens through their B cell receptors and contribute to autoimmunity by presenting autoantigens to pathogenic T cells providing T cell help, production of pro-inflammatory cytokines and formation of tertiary lymphoid structures, a process termed neo-organogenesis. Consequently, B cell-targeted therapies have emerged as an effective therapy in patients with autoimmune diseases even in diseases that are believed to be mainly mediated by T cells, such as rheumatoid arthritis and multiple sclerosis (66–69).

## Efficacy of B Cell Depleting Therapy With Rituximab in Refractory Chronic Recurrent Uveitis Associated With VKH Disease

Rituximab is a chimeric mouse/human monoclonal antibody against the pan B cell marker CD20. CD20 is a transmembrane protein expressed on naive and memory B cells, but not on stem cells or fully differentiated plasma cells. Rituximab has emerged as an effective therapy in patients with autoimmune diseases refractory to conventional immunosuppression, with the rationale of depleting pathogenic B cells (66–69). The efficacy of anti-CD20-mediated B cell depletion in treating autoimmune diseases supports an important role for B cells in the development and propagation of these diseases.

Rituximab was reported to be effective in three individual cases of resistant uveitis associated with VKH disease (70–72). We presented the first series and long-term follow-up study of 9 patients with refractory chronic recurrent granulomatous uveitis associated with VKH disease treated with rituximab. All patients were resistant to conventional immunosuppressive therapy and TNF-α blockers. In this group of patients, rituximab made a significant contribution to control inflammation. Adjuvant rituximab allowed the daily immunosuppressive doses to be reduced (73). Similarly, Bolletta et al. (74) demonstrated the efficacy of rituximab therapy in 5 patients with refractory uveitis associated with VKH disease. The good response after rituximab therapy suggests an important role of B cells in the pathogenesis of chronic recurrent uveitis associated with VKH disease.

## Conclusions

In conclusion, VKH disease is a true autoimmune disorder that has become an example in the field with the demonstration that the combination of research efforts in basic immunology, the use of preclinical animal models and critical attitudes to association and clinical case report studies leads to the cure of initial-onset acute VKH disease, possible by preventing autoimmunization. In addition, for VKH patients who have progressed into chronic disease, treatment with rituximab to control the damage caused by autoantibodies and by B lymphocytes acting as antigen-presenting cells to pathogenic T cells, represents a therapeutic modality that needs broad attention. Both types of treatments, early aggressive immunosuppression preventing autoimmunization (20) and rituximab in progressed VKH disease are not antigen-specific. Thanks to new developments toward antigen-specific immune tolerance induction with mRNAs encoding specific autoantigens (75), we may now also be on the verge to additional progress, also for VKH disease.

## Author Contributions

AA wrote the manuscript. SS, GO, and JV critically edited the manuscript. All authors contributed to the manuscript and approved the final submission.

## Funding

This work was supported by King Saud University through Vice Deanship of Research Chair, Dr. Nasser Al-Rashid Research Chair in Ophthalmology (AA). At KU Leuven, support was provided by the Research Foundation of Flanders (FWO-Vlaanderen) and C1 funding 2017-2023.

## Conflict of Interest

The authors declare that the research was conducted in the absence of any commercial or financial relationships that could be construed as a potential conflict of interest.

## Publisher's Note

All claims expressed in this article are solely those of the authors and do not necessarily represent those of their affiliated organizations, or those of the publisher, the editors and the reviewers. Any product that may be evaluated in this article, or claim that may be made by its manufacturer, is not guaranteed or endorsed by the publisher.

## References

[1] Yamaki K Gocho K Hayakawa K Kondo I Sakuragi S. Tyrosinase family proteins are antigens specific to Vogt-Koyanagi-Harada disease. J Immunol. (2000) 165:7323–9. 10.4049/jimmunol.165.12.7323 11120868

[2] Yamaki K Kondo I Nakamura H Miyano M Konno S Sakuragi S. Ocular and extraocular inflammation induced by immunization of tyrosinase related protein 1 and 2 in Lewis rats. Exp Eye Res. (2000) 71:361–9. 10.1006/exer.2000.0893 10995557

[3] Gocho K Kondo I Yamaki K. Identification of autoreactive T cells in Vogt-Koyanagi-Harada disease. Invest Ophthalmol Vis Sci. (2001) 42:2004–9. 11481264

[4] Abu El-Asrar AM Alsarhani W Alzubaidi A Gikandi PW. Effect of immunosuppressive therapy on ocular blood flow in initial-onset acute uveitis associated with Vogt-Koyanagi-Harada disease. Acta Ophthalmol. (2021). 10.1111/aos.14842 [Epub ahead of print]. 33719161

[5] Nakao K Abematsu N Mizushima Y Sakamoto T. Optic disc swelling in Vogt-Koyanagi-Harada disease. Invest Ophthalmol Vis Sci. (2012) 53:1917–22. 10.1167/iovs.11-8984 22408010

[6] Yang P Ren Y Li B Fang W Meng Q Kijlstra A. Clinical characteristics of Vogt-Koyanagi-Harada syndrome in Chinese patients. Ophthalmology. (2007) 114:606–14. 10.1016/j.ophtha.2006.07.040 17123618

[7] Fang W Yang P. Vogt-Koyanagi-Harada syndrome. Curr Eye Res. (2008) 33:517–23. 10.1080/02713680802233968 18600484

[8] Abu El-Asrar AM Al Tamimi M Hemachandran S Al-Mezaine HS Al-Muammar A Kangave D. Prognostic factors for clinical outcomes in patients with Vogt-Koyanagi-Harada disease treated with high-dose corticosteroids. Acta Ophthalmol. (2013) 91:e486–93. 10.1111/aos.12127 23575246

[9] AlBloushi AF Alfawaz AM AlZaid A Alsalamah AK Gikandi PW Abu El-Asrar AM. Incidence, risk factors and surgical outcomes of cataract among patients with Vogt-Koyanagi-Harada disease. Ocul Immunol Inflamm. (2021) 29:128–36. 10.1080/09273948.2019.1668430 31638886

[10] Abu El-Asrar AM Hemachandran S Al-Mezaine HS Kangave D Al-Muammar AM. The outcomes of mycophenolate mofetil therapy combined with systemic corticosteroids in acute uveitis associated with Vogt-Koyanagi-Harada disease. Acta Ophthalmol. (2012) 90:e603–8. 10.1111/j.1755-3768.2012.02498.x 22971163

[11] Abu El-Asrar AM Dosari M Hemachandran S Gikandi PW Al-Muammar A. Mycophenolate mofetil combined with systemic corticosteroids prevents progression to chronic recurrent inflammation and development of 'sunset glow fundus' in initial-onset acute uveitis associated with Vogt-Koyanagi-Harada disease. Acta Ophthalmol. (2017) 95:85–90. 10.1111/aos.13189 27535102

[12] Nakamura S Nakazawa M Yoshioka M Nagano I Nakamura H Onodera J. Melanin-laden macrophages in cerebrospinal fluid in Vogt-Koyanagi-Harada syndrome. Arch Ophthalmol. (1996) 114:1184–8. 10.1001/archopht.1996.01100140384003 8859075

[13] Moorthy RS Inomata H Rao NA. Vogt-Koyanagi-Harada syndrome. Surv Ophthalmol. (1995) 39:265–92. 10.1016/S0039-6257(05)80105-5 7725227

[14] Abu El-Asrar AM Al Mudhaiyan T Al Najashi AA Hemachandran S Hariz R Mousa A. Chronic recurrent Vogt–Koyanagi–Harada disease and development of ‘sunset glow fundus' predict worse retinal sensitivity. Ocul Immunol Inflamm. (2017) 25:475–85. 10.3109/09273948.2016.1139730 27003480

[15] Fang W Zhou H Yang P Huang X Wang L Kijlstra A. Longitudinal quantification of aqueous flare and cells in Vogt-Koyanagi-Harada disease. Br J Ophthalmol. (2008) 92:182–5. 10.1136/bjo.2007.128967 17965105

[16] Bacsal K Wen DS Chee S-P. Concomitant choroidal inflammation during anterior segment recurrence in Vogt-Koyanagi-Harada disease. Am J Ophthalmol. (2008) 145:480–6. 10.1016/j.ajo.2007.10.012 18191100

[17] Inomata H Sakamoto T. Immunohistochemical studies of Vogt-Koyanagi-Harada disease with sunset sky fundus. Curr Eye Res. (1990) 9(Suppl):35–40. 10.3109/02713689008999417 1974489

[18] Lai TY Chan RP Chan CK Lam DS. Effects of the duration of initial oral corticosteroid treatment on the recurrence of inflammation in Vogt-Koyanagi-Harada disease. Eye. (2009) 23:543–8. 10.1038/eye.2008.89 18369377

[19] Kawaguchi T Horie S Bouchenaki N Ohno-Matsui K Mochizuki M Herbort CP. Suboptimal therapy controls clinically apparent disease but not subclinical progression of Vogt-Koyanagi-Harada disease. Int Ophthalmol. (2010) 30:41–50. 10.1007/s10792-008-9288-1 19151926

[20] Opdenakker G Abu El-Asrar A Van Damme J. Remnant epitopes generating autoimmunity: From model to useful paradigm. Trends Immunol. (2020) 41:367–78. 10.1016/j.it.2020.03.004 32299652

[21] Finckh A Liang MH van Herckenrode CM de Pablo P. Long-term impact of early treatment on radiographic progression in rheumatoid arthritis: a meta-analysis. Arthritis Rheum. (2006) 55:864–72. 10.1002/art.22353 17139662

[22] van Nies JA Krabben A Schoones JW Huizinga TW Kloppenburg M van der Helm-van Mil AH. What is the evidence for the presence of a therapeutic window of opportunity in rheumatoid arthritis? A systematic literature review. Ann Rheum Dis. (2014) 73:861–70. 10.1136/annrheumdis-2012-203130 23572339

[23] Herbort CP Jr Abu El Asrar AM Takeuchi M Pavésio CE Couto C Hedayatfar A. Catching the therapeutic window of opportunity in early initial-onset Vogt-Koyanagi-Harada uveitis can cure the disease. Int Ophthalmol. (2019) 39:1419–25. 10.1007/s10792-018-0949-4 29948499

[24] Keino H Goto H Usui M. Sunset glow fundus in Vogt-Koyanagi-Harada disease with or without chronic ocular inflammation. Graefes Arch Clin Exp Ophthalmol. (2002) 240:878–82. 10.1007/s00417-002-0538-z 12397437

[25] Keino H Goto H Mori H Iwasaki T Usui M. Association between severity of inflammation in CNS and development of sunset glow fundus in Vogt-Koyanagi-Harada disease. Am J Ophthalmol. (2006) 141:1140–2. 10.1016/j.ajo.2006.01.017 16765691

[26] Chee S-P Jap A Bacsal K. Spectrum of Vogt-Koyanagi-Harada disease in Singapore. Int Ophthalmol. (2007) 27:137–42. 10.1007/s10792-006-9009-6 17103022

[27] Tugal-Tutkun I Ozyazgan Y Akova YA Sullu Y Akyol N Soylu M. The spectrum of Vogt-Koyanagi-Harada disease in Turkey: VKH in Turkey. Int Ophthalmol. (2007) 27:117–23. 10.1007/s10792-006-9001-1 16957877

[28] Sakata VM da Silva FT Hirata CE Marin ML Rodrigues H Kalil J. High rate of clinical recurrence in patients with Vogt-Koyanagi-Harada disease treated with early high-dose corticosteroids. Graefes Arch Clin Exp Ophthalmol. (2015) 253:785–90. 10.1007/s00417-014-2904-z 25592477

[29] Nakayama M Keino H Watanabe T Okada AA. Clinical features and visual outcomes of 111 patients with new-onset acute Vogt-Koyanagi-Harada disease treated with pulse intravenous corticosteroids. Br J Ophthalmol. (2019)103:274–8. 10.1136/bjophthalmol-2017-311691 29666121

[30] Cuchacovich M Solanes F Díaz G Cermenati T Avila S Verdaguer J. Comparison of the clinical efficacy of two different immunosuppressive regimens in patients with chronic Vogt-Koyanagi-Harada disease. Ocul Immunol Inflamm. (2010) 18:200–7. 10.3109/09273941003587541 20482399

[31] Kim SJ Yu HG. The use of low-dose azathioprine in patients with Vogt-Koyanagi-Harada disease. Ocul Immunol Inflamm. (2007) 15:381–7. 10.1080/09273940701624312 17972222

[32] Paredes I Ahmed M Foster CS. Immunomodulatory therapy for Vogt-Koyanagi-Harada patients as first-line therapy. Ocul Immunol Inflamm. (2006) 14:87–90. 10.1080/09273940500536766 16597537

[33] Lin Oo EE Chee SP Wong KKY Htoon HM. Vogt-Koyanagi-Harada disease managed with immunomodulatory therapy within 3 months of disease onset. Am J Ophthalmol. (2020) 220:37–44. 10.1016/j.ajo.2020.07.036 32738228

[34] Beller DI Unanue ER. Evidence that thymocytes require at least two distinct signals to proliferate. J Immunol. (1979) 123:2890–3. 315434

[35] Goldberg AC Yamamoto JH Chiarella JM Marin ML Sibinelli M Neufeld R. HLA-DRB1^*^0405 is the predominant allele in Brazilian patients with Vogt-Koyanagi-Harada disease. Hum Immunol. (1998) 59:183–8. 10.1016/S0198-8859(97)00265-6 9548078

[36] Damico FM Cunha-Neto E Goldberg AC Iwai LK Marin ML Hammer J. T-cell recognition and cytokine profile induced by melanocyte epitopes in patients with HLA-DRB1^*^0405-positive and -negative Vogt-Koyanagi-Harada uveitis. Invest Ophthalmol Vis Sci. (2005) 46:2465–71. 10.1167/iovs.04-1273 15980237

[37] Abad S Wieërs G Colau D Wildmann C Delair E Dhote R. Absence of recognition of common melanocytic antigens by T cells isolated from the cerebrospinal fluid of a Vogt-Koyanagi-Harada patient. Mol Vis. (2014) 20:956–69. 24991188 PMC4077848

[38] Greco A Fusconi M Gallo A Turchetta R Marinelli C Macri GF. Vogt-Koyanagi-Harada syndrome. Autoimmun Rev. (2013) 12:1033–8. 10.1016/j.autrev.2013.01.004 23567866

[39] Sood AB O'Keefe G Bui D Jain N. Vogt-Koyanagi-Harada disease associated with hepatitis B vaccination. Ocul Immunol Inflamm. (2019) 27:524–7. 10.1080/09273948.2018.1483520 29953303

[40] Touitou V Bodaghi B Cassoux N Tran TH Rao NA Cacoub P. Vogt-Koyanagi-Harada disease in patients with chronic hepatitis C. Am J Ophthalmol. (2005) 140:949–52. 10.1016/j.ajo.2005.06.020 16310490

[41] Bassili SS Peyman GA Gebhardt BM Daun M Ganiban GJ Rifai A. Detection of Epstein-Barr virus DNA by polymerase chain reaction in the vitreous from a patient with Vogt-Koyanagi-Harada syndrome. Retina. (1996) 16:160–1. 10.1097/00006982-199616020-00013 8724962

[42] Hotta Y Hayakawa M Kawano H Sakuma H Momose T Ohkoshi K. Analysis of herpes virus group (DNA) from cerebrospinal fluid in Vogt-Koyanagi-Harada disease. Ocul Immunol Inflamm. (1996) 4:99–103. 10.3109/09273949609079639 22827414

[43] Andrei G Van Loon E Lerut E Victoor J Meijers B Bammens B. Persistent primary cytomegalovirus infection in a kidney transplant recipient: Multi-drug resistant and compartmentalized infection leading to graft loss. Antiviral Res. (2019) 168:203–9. 10.1016/j.antiviral.2019.06.004 31212020

[44] Oldstone MB. Molecular mimicry, microbial infection, and autoimmune disease: evolution of the concept. Curr Top Microbiol Immunol. (2005) 296:1–17. 10.1007/3-540-30791-5_1 16329189 PMC7120699

[45] Sugita S Takase H Kawaguchi T Taguchi C Mochizuki M. Cross-reaction between tyrosinase peptides and cytomegalovirus antigen by T cells from patients with Vogt-Koyanagi-Harada disease. Int Ophthalmol. (2007) 27:87–95. 10.1007/s10792-006-9020-y 17253112

[46] Abu El-Asrar AM Berghmans N Al-Obeidan SA Mousa A Opdenakker G Van Damme J. The cytokine interleukin-6 and the chemokines CCL20 and CXCL13 are novel biomarkers of specific endogenous uveitic entities. Invest Ophthalmol Vis Sci. (2016) 57:4606–13. 10.1167/iovs.16-19758 27603722

[47] El-Asrar AMA Berghmans N Al-Obeidan SA Gikandi PW Opdenakker G Van Damme J. Differential CXC and CX3C Chemokine expression profiles in aqueous humor of patients with specific endogenous uveitic entities. Invest Ophthalmol Vis Sci. (2018) 59:2222–8. 10.1167/iovs.17-23225 29715366

[48] Abu El-Asrar AM Berghmans N Al-Obeidan SA Gikandi PW Opdenakker G Van Damme J. Local cytokine expression profiling in patients with specific autoimmune uveitic entities. Ocul Immunol Inflamm. (2020) 28:453–62. 10.1080/09273948.2019.1604974 31161935

[49] Abu El-Asrar AM Berghmans N Al-Obeidan SA Gikandi PW Opdenakker G Van Damme J. The CC chemokines CCL8, CCL13 and CCL20 are local inflammatory biomarkers of HLA-B27-associated uveitis. Acta Ophthalmol. (2019) 97:e122–8. 10.1111/aos.13835 30242977

[50] Abu El-Asrar AM Berghmans N Al-Obeidan SA Gikandi PW Opdenakker G Van Damme J. Expression of interleukin (IL)-10 family cytokines in aqueous humour of patients with specific endogenous uveitic entities: elevated levels of IL-19 in human leucocyte antigen-B27-associated uveitis. Acta Ophthalmol. (2019) 97:e780–4. 10.1111/aos.14039 30761764

[51] Abu El-Asrar AM Berghmans N Al-Obeidan SA Gikandi PW Opdenakker G Van Damme J. Soluble cytokine receptor levels in aqueous humour of patients with specific autoimmune uveitic entities: sCD30 is a biomarker of granulomatous uveitis. Eye. (2020) 34:1614–23. 10.1038/s41433-019-0693-7 31804623 PMC7608430

[52] Legler DF Loetscher M Roos RS Clark-Lewis I Baggiolini M Moser B. B cell-attracting chemokine 1, a human CXC chemokine expressed in lymphoid tissues, selectively attracts B lymphocytes via BLR1/CXCR5. J Exp Med. (1998) 187:655–60 10.1084/jem.187.4.655 PMC2212150 9463416

[53] Gunn MD Ngo VN Ansel KM Ekland EH Cyster JG Williams LT. B-cell-homing chemokine made in lymphoid follicles activates Burkitt's lymphoma receptor-1. Nature. (1998) 391:799–803. 10.1038/35876 9486651

[54] Bugatti S Manzo A Vitolo B Benaglio F Binda E Scarabelli M. High expression levels of the B cell chemoattractant CXCL13 in rheumatoid synovium are a marker of severe disease. Rheumatology. (2014) 53:1886–95. 10.1093/rheumatology/keu163 24764267

[55] Greisen SR Schelde KK Rasmussen TK Kragstrup TW Stengaard-Pedersen K Hetland ML. CXCL13 predicts disease activity in early rheumatoid arthritis and could be an indicator of the therapeutic 'window of opportunity'. Arthritis Res Ther. (2014) 16:434. 10.1186/s13075-014-0434-z 25249397 PMC4201737

[56] Schiffer L Worthmann K Haller H Schiffer M. CXCL13 as a new biomarker of systemic lupus erythematosus and lupus nephritis – from bench to bedside? Clin Exp Immunol. (2014) 179:85–9. 10.1111/cei.12439 25138065 PMC4260900

[57] Wong CK Wong PTY Tam LS Li EK Chen DP Lam CWK. Elevated production of B cell chemokine CXCL13 is correlated with systemic lupus erythematosus disease activity. J Clin Immunol. (2010) 30:45–52. 10.1007/s10875-009-9325-5 19774453

[58] Sellebjerg F Börnsen L Khademi M Krakauer M Olsson T Frederiksen JL. Increased cerebrospinal fluid concentrations of the chemokine CXCL13 in active MS. Neurology. (2009) 73:2003–10. 10.1212/WNL.0b013e3181c5b457 19996075

[59] Krumbholz M Theil D Cepok S Hemmer B Kivisäkk P Ransohoff RM. Chemokines in multiple sclerosis: CXCL12 and CXCL13 up-regulation is differentially linked to CNS immune cell recruitment. Brain. (2006) 129(Pt 1):200–11. 10.1093/brain/awh680 16280350

[60] Shiao YM Lee CC Hsu YH Huang SF Lin CY Li LH Fann CS. Ectopic and high CXCL13 chemokine expression in myasthenia gravis with thymic lymphoid hyperplasia. J Neuroimmunol. (2010) 221:101–6. 10.1016/j.jneuroim.2010.02.013 20223524

[61] Klimatcheva E Pandina JT Rilly C Torno S Bussler H Scrivens M. CXCL13 antibody for the treatment of autoimmune disorders. BMC Immunol. (2015) 16:6. 10.1186/s12865-015-0068-1 25879435 PMC4329654

[62] Lahiri A Pochard P Le Pottier L Tobón GJ Bendaoud B Youinou P. The complexity of the BAFF TNF-family members: implications for autoimmunity. J Autoimmun. (2012) 39:189–98. 10.1016/j.jaut.2012.05.009 22749832

[63] Aziz HA Flynn HW Jr Young RC Davis JL Dubovy SR. Sympathetic ophthalmia: clinicopathologic correlation in a consecutive case series. Retina. (2015) 35:1696–703. 10.1097/IAE.0000000000000506 25719985 PMC4514550

[64] Abu El-Asrar AM Struyf S Van den Broeck C Van Damme J Opdenakker G Geboes K. Expression of chemokines and gelatinase B in sympathetic ophthalmia. Eye. (2007) 21:649–57. 10.1038/sj.eye.6702342 16601741

[65] Shah DN Piacentini MA Burnier MN McLean IW Nussenblatt RB Chan CC. Inflammatory cellular kinetics in sympathetic ophthalmia a study of 29 traumatized (exciting) eyes. Ocul Immunol Inflamm. (1993) 1:255–62. 10.3109/09273949309085026 22822781

[66] Chan CC Palestine AG Kuwabara T Nussenblatt RB. Immunopathologic study of Vogt-Koyanagi-Harada syndrome. Am J Ophthalmol. (1988) 105:607–11. 10.1016/0002-9394(88)90052-9 3259837

[67] Franks SE Getahun A Hogarth PM Cambier JC. Targeting B cells in treatment of autoimmunity. Curr Opin Immunol. (2016) 43:39–45. 10.1016/j.coi.2016.09.003 27718447 PMC5127449

[68] Hofmann K Clauder AK Manz RA. Targeting B cells and plasma cells in autoimmune diseases. Front Immunol. (2018) 9:835. 10.3389/fimmu.2018.00835 29740441 PMC5924791

[69] Musette P Bouaziz JDB. Cell Modulation Strategies in Autoimmune Diseases: New Concepts. Front Immunol. (2018) 9:622. 10.3389/fimmu.2018.00622 29706952 PMC5908887

[70] Caso F Rigante D Vitale A Costa L Bascherini V Latronico E. Long-lasting uveitis remission and hearing loss recovery after rituximab in Vogt-Koyanagi-Harada disease. Clin Rheumatol. (2015) 34:1817–20. 10.1007/s10067-014-2781-1 25224382

[71] Umran RMR Shukur ZYH. Rituximab for sight-threatening refractory pediatric Vogt-Koyanagi-Harada disease. Mod Rheumatol. (2018) 28:197–9. 10.3109/14397595.2015.1071234 26154298

[72] Dolz-Marco R Gallego-Pinazo R Díaz-Llopis M. Rituximab in refractory Vogt-Koyanagi-Harada disease. J Ophthalmic Inflamm Infect. (2011) 1:177–80. 10.1007/s12348-011-0027-9 21744181 PMC3223341

[73] Abu El-Asrar AM Dheyab A Khatib D Struyf S Van Damme J Opdenakker G. Efficacy of B cell depletion therapy with rituximab in refractory chronic recurrent uveitis associated with Vogt-Koyanagi-Harada disease. Ocul Immunol Inflamm. (2020) 29:1–8. 10.1080/09273948.2020.1820531 32990482

[74] Bolletta E Gozzi F Mastrofilippo V Pipitone N De Simone L Croci S. Efficacy of rituximab treatment in Vogt-Koyanagi-Harada disease poorly controlled by traditional immunosuppressive treatment. Ocul Immunol Inflamm. (2021). 10.1080/09273948.2021.1880604 [Epub ahead of Print]. 33793383

[75] Krienke C Kolb L Diken E Streuber M Kirchhoff S Bukur T. A noninflammatory mRNA vaccine for treatment of experimental autoimmune encephalomyelitis. Science. (2021) 371:145–53. 10.1126/science.aay3638 33414215

